# The role of galectins in mediating the adhesion of circulating cells to vascular endothelium

**DOI:** 10.3389/fimmu.2024.1395714

**Published:** 2024-05-22

**Authors:** Joseph Souchak, Norhan B. B. Mohammed, Lee Seng Lau, Charles J. Dimitroff

**Affiliations:** ^1^ Department of Cellular and Molecular Medicine, Herbert Wertheim College of Medicine, Florida International University, Miami, FL, United States; ^2^ Department of Medical Biochemistry, Faculty of Medicine, South Valley University, Qena, Egypt

**Keywords:** galectin-3, galectin-8, galectin-9, endothelial cells, cell adhesion

## Abstract

Vascular cell adhesion is a complex orchestration of events that commonly feature lectin–ligand interactions between circulating cells, such as immune, stem, and tumor cells, and endothelial cells (ECs) lining post-capillary venules. Characteristically, circulating cell adherence to the vasculature endothelium is initiated through interactions between surface sialo-fucosylated glycoprotein ligands and lectins, specifically platelet (P)- or endothelial (E)-selectin on ECs or between leukocyte (L)-selectin on circulating leukocytes and L-selectin ligands on ECs, culminating in circulating cell extravasation. This lectin–ligand interplay enables the migration of immune cells into specific tissue sites to help maintain effective immunosurveillance and inflammation control, the homing of stem cells to bone marrow or tissues in need of repair, and, unfortunately, in some cases, the dissemination of circulating tumor cells (CTCs) to distant metastatic sites. Interestingly, there is a growing body of evidence showing that the family of β-galactoside-binding lectins, known as galectins, can also play pivotal roles in the adhesion of circulating cells to the vascular endothelium. In this review, we present contemporary knowledge on the significant roles of host- and/or tumor-derived galectin (Gal)-3, -8, and -9 in facilitating the adhesion of circulating cells to the vascular endothelium either directly by acting as bridging molecules or indirectly by triggering signaling pathways to express adhesion molecules on ECs. We also explore strategies for interfering with galectin-mediated adhesion to attenuate inflammation or hinder the metastatic seeding of CTCs, which are often rich in galectins and/or their glycan ligands.

## 1 Introduction

It is well established that the rapid and tissue-specific homing of circulating immune or tumor cells relies on a series of interactions with the vascular endothelium, preceding transendothelial migration (also known as diapedesis or extravasation), chemotaxis, and adaptation to the tissue microenvironment (1, 2). The critical initial phase in this process is vascular cell adhesion, where the circulating cells adhere to the endothelial cells (ECs) lining blood vessels, primarily within distinct post-capillary venular segments (3, 4). Vascular cell adhesion plays a crucial role in various physiological processes, including embryonic development, immune response regulation, tissue repair, and inflammation control (5). Unsurprisingly, dysregulated vascular cell adhesion often directly contributes to diverse pathological conditions, such as psoriasis, rheumatoid arthritis, impaired wound healing, asthma, patient transplant rejection, and cancer metastasis (6).

The adhesion of circulating cells to the vascular endothelium involves several sequential steps, including cell tethering, rolling, and firm adhesion (7). These steps are orchestrated by surface molecules expressed on both circulating cells and ECs (8–10). Considering the fact that most cell surface and secreted molecules are glycosylated, it becomes evident that glycan–glycan binding protein interactions are pervasive in controlling, or at least tuning, human biological activities, and when dysregulated, can lead to cellular dysfunction and disease development (11). Glycan-binding proteins (also known as lectins) comprise a diverse group of proteins categorized into 25 distinct families, each of which binds discrete carbohydrate structures (12). C-type lectins (including selectins), S-type Lectins (galectins), and I-type lectins (sialic acid-binding immunoglobin-like lectins; siglecs) are three large families of mammalian lectins known to play crucial roles as mediators of vascular cell adhesion (13).

Galectin-mediated vascular cell adhesion is currently recognized as a significant contributing molecular mechanism (14). Galectins can facilitate both homotypic and heterotypic cell adhesion in a calcium-independent manner (11, 15–17). Galectin (Gal)-3, -8, and -9, in particular, have emerged as key players in mediating the adhesion between circulating cells and the vascular endothelium. Consequently, strategic inhibition of Gal-3, -8, and -9 expression or function has been documented to reduce vascular cell adhesion (18–24). In this review, we will explore the roles of Gal-3, -8, and -9 in vascular cell adhesion and their increasingly recognized importance in various aspects of autoimmunity, infectious diseases, and malignancy.

## 2 Canonical mechanisms of glycan-mediated cell adherence to vascular endothelium

Molecular mechanisms of circulating cell adherence to vascular ECs involve expression and presentation of lectins and cell surface glycosylation that serve as lectin ligands (25, 26). These glycosylated lectin ligands presented by circulating cells or ECs help initiate cell adhesion through binding interactions with lectins expressed on ECs or circulating cells, respectively, with a high degree of specificity (27).

Selectins are a group of calcium-dependent (C)-type lectins that are characteristically involved in vascular cell adhesion (28, 29). There are three members within the selectin family: Endothelial (E), Platelet (P), and Leukocyte (L)-selectin (30). Each selectin is comprised of an N-terminal carbohydrate recognition or lectin domain (CRD), epidermal growth factor (EGF)-like domain, a variable number of consensus repeats (CRs), a single transmembrane domain, and a short cytoplasmic tail (28, 31). Selectins play a crucial role in the initial stages of cell adhesion, namely in the “tethering and rolling” process, which is critical for the capture of circulating cells to the post-capillary venules that initiates diapedesis of circulating cells into tissues (2). Selectins extend from the cell surface, allowing for the CRD to recognize glycan ligands, notably sialyl Lewis X (sLe^X^) or A (sLe^A^), presented by surface glycoproteins and glycosphingolipids (2).

E-selectin (CD62E) expression is constitutive on dermal and bone marrow post-capillary venule ECs, though its levels on vascular ECs in other tissues are shown to be regulated by TNF-α, IL-1β, or bacterial lipopolysaccharides (2, 29, 32, 33).

P-selectin (CD62P) is stored in specialized secretory organelles in ECs, called Weibel-Palade bodies, and in α-granules of circulating platelets, and can be quickly translocated to the cell surface upon activation through mediators, such histamine, thrombin, or other components of the complement system (34, 35). Furthermore, other P-selectin ligands found on hematopoietic stem cells include CD24 and CD34 (36, 37).

L-selectin (CD62L) is variably expressed on the microvilli of circulating leukocytes depending on the state of differentiation and characteristically involved in lymphocyte homing to secondary lymphoid tissues and in facilitating leukocyte migration to sites of inflammation (38–40).

SLe^X^ and sLe^A^ are canonical glycan structures required for binding to selectins and are widely recognized as key molecular features in vascular cell adhesion due to their noted expression on circulating stem cells, leukocytes, CTCs, and ECs (41). SLe^A^ is predominantly found on CTCs of epithelial origin (42), whereas sLe^X^ expression is observed on a variety of circulating cells, including CTCs (43), granulocytes (44, 45), lymphocytes (46, 47), monocytes (46), dendritic cells (48), and hematopoietic stem cells (49). SLe^X^ and sLe^A^ can interact with E-selectin to mediate the initial first step of vascular cell adhesion, “tethering and rolling,” for circulating cells on the vascular endothelium that prompts firm adhesion and eventually transendothelial migration (2, 50–54). Similarly, SLe^X^-bearing surface glycoproteins, primarily P-selectin glycoprotein ligand-1 (PSGL-1) on leukocytes, for example, are crucial for vascular cell adhesion through their interaction with P-selectin, requiring tyrosine sulfation adjacent to these glycoproteins at the PSGL-1 terminus for effective binding (30, 55, 56). SLe^X^ moieties, present on glycan ligands like Sgp200, GlyCAM-1, MAdCAM-1, endoglycan, endomucin, and podocalyxin-like protein (PCLP) on ECs lining high endothelial venules, bind L-selectin-bearing lymphocytes and are further modified by GlcNAc-6-O-sulfotransferases on CD34 O-glycans to generate 6-sulfo sLe^X^, enhancing L-selectin binding (11, 40, 57).. Furthermore, presentation of sLe^X^ has been shown on a specialized glycoform of CD44, known as HCELL (hematopoietic stem cell E/L-selectin ligand) expressed by normal and malignant hematopoietic stem cells (41, 58, 59), CD34 as a ligand for E/P-selecting on hematopoietic cells (36), CD43 (60, 61) and PSGL-1, which is found on most leukocytes (62).

## 3 Galectin-mediated cell adherence to vascular endothelium

**Figure 1:**
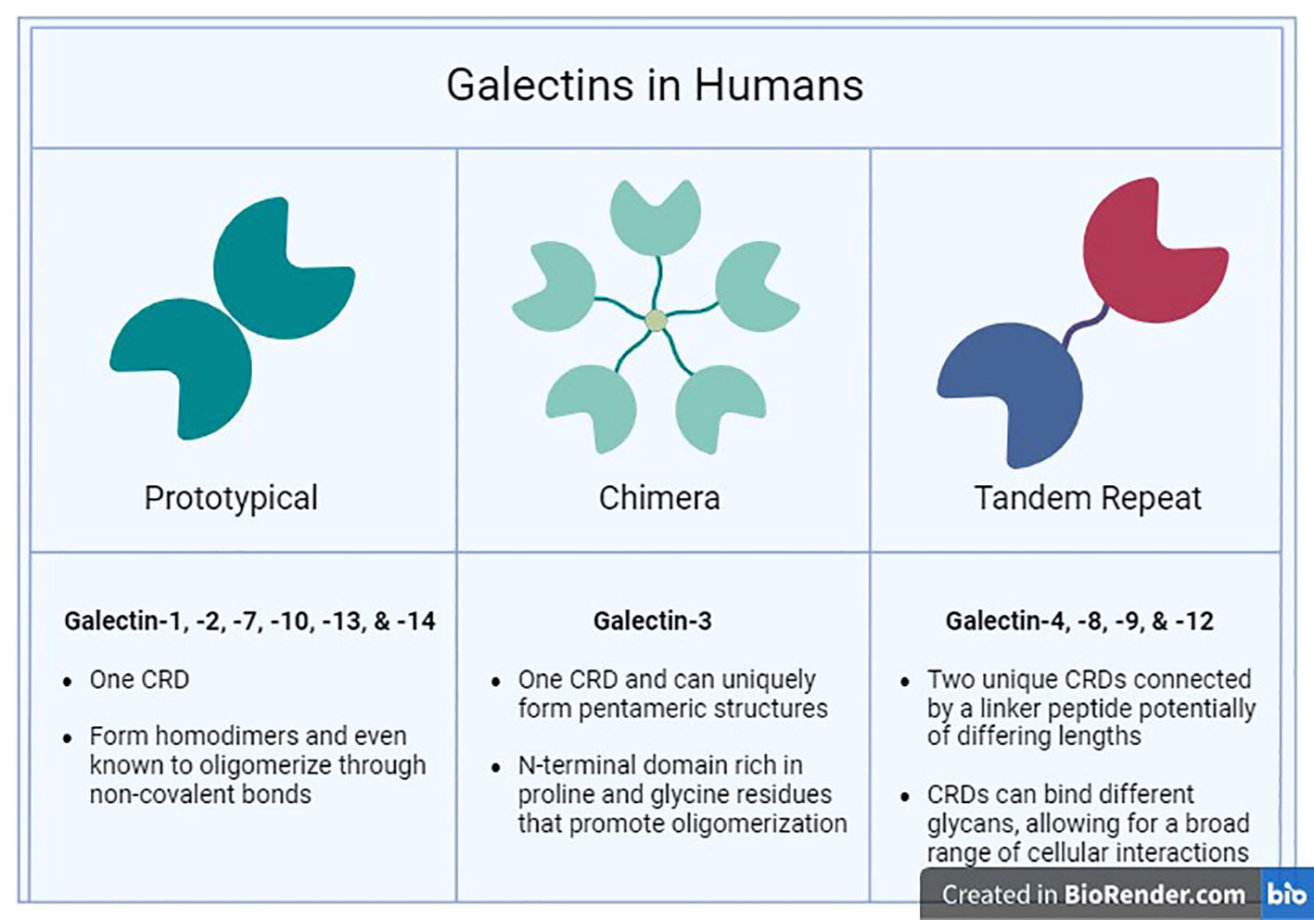
Human Galectins. The three major families (Prototypical, Chimera, and Tandem Repeat) of galectins found in humans are categorized by their structural characteristics and ligand selectivity properties.

Galectins are a family of β-galactoside-binding lectins produced within the cytosol and extruded into the extracellular milieu through non-classical secretory routes due to lack of signal sequence (63–66). Galectins are categorized, based on their molecular structure, into three types: prototypical, chimeric, and tandem repeat galectins (**Figure 1**) (67). The prototypical galectins (Gal-1, -2, -5, -7, -10, -11, -13, -14, -15, and -16) have a single CRD, designed to directly interact with β-galactoside-bearing glycans and can assemble into homodimers (68). The chimeric galectin family contains only one member (Gal-3), which has a single CRD linked to an N-terminal domain (NTD) (67, 69). The tandem repeat galectins (Gal-4, -6, -8, -9, and -12) comprise two distinct CRDs with unique binding preferences connected together by a linker peptide crucial for their complete functionality (70).

Galectin functional expression varies enormously based on the tissue type and cellular/subcellular location, in which they function as intra- or extracellular modifiers, regulating a plethora of cellular activities such as cell differentiation, proliferation, apoptosis, and immune responses (11). Moreover, some galectins are involved in tumor progression through regulating intracellular oncogenic signaling, immune evasion, angiogenesis, and circulating tumor cell (CTC) survival, lodging, and proliferation in metastatic tissues (69, 71, 72). While adhesion molecules that mediate circulating cell interaction with vascular endothelium typically reside on the cell surface, some galectins have been suggested to function as soluble adhesion molecules that facilitate cross-linking of cell surface glycoconjugates (73). There is a large body of evidence emphasizing the importance of Gal-3, -8, and -9 in mediating circulating cells interactions with ECs, which we will delve into further in this section.

### 3.1 Gal-3

Gal-3 was first recognized in the early 1980s as a 35kDa β-galactoside-binding protein on murine peritoneal macrophages and referred to as Mac-2, IgE-binding protein, L-29, and L-31 (11, 27). Gal-3 is encoded by a single gene (*LGALS3*), located on chromosome 14, locus q21-q22, consisting of six exons and five introns (74). Gal-3 is synthesized by free cytosolic ribosomes and can translocate into the nucleus or be secreted into the extracellular space (75).

A hallmark glycan ligand of Gal-3 is a poly-N-acetyllactosamine (poly-LacNAc), which consists of repeating Galβ1,4GlcNAcβ1,3 disaccharides (76). Another ligand for Gal-3 is N,N′-diacetyllactosamine (GalNAcβ1,4GlcNAc or LacdiNAc) (77) found on a select group of N- and/or O-linked glycoproteins (76). Numerous studies have indicated that the interaction between Gal-3 and its ligands is influenced by the presence of terminal sialylation (78). In fact, α2,6 sialylation of terminal galactose residues on N-glycans via β-galactoside α2,6 sialyltransferase 1 (ST6GAL1) or of N-acetylgalactosamine on O-glycans by N-acetylgalactosamine α2,6 sialyltransferase 1 (ST6GALNAC1) significantly downregulates Gal-3 binding to glycan ligands (79, 80).

The characteristic structure of Gal-3 allows two forms of oligomerization: type N self-association mediated by the NTDs resulting in the formation of pentameric complexes, and type C self-association through the CRDs to form dimers or oligomers (81, 82). Extracellular Gal-3 oligomers are implicated in the adhesive and migratory activities of normal and cancer cells through binding interactions between glycosylated ligands present either on cell surfaces or in the ECM, including fibronectin, laminin, collagen IV, and elastin (83). In terms of vascular cell adhesion, Gal-3 facilitates the attachment of diverse cell types to ECs, initiating the homing of these cells to various tissues throughout the body, as elaborated below (84–86).

#### 3.1.1 Gal-3-mediated immune cell adhesion to vascular endothelium

Mounting evidence supports Gal-3’s important role in coordinating the trafficking of leukocytes toward sites of injury or inflammation (84). Early *in vivo* studies utilizing a streptococcal pneumonia mouse model explore the role of Gal-3 as a modulator of neutrophil-mediated innate immunity (73). The authors report that Gal-3 is released extracellularly into alveolar spaces infected by *Streptococcus pneumoniae* at the onset of neutrophil extravasation (73). Immunohistochemistry analysis further showed an accumulation of Gal-3 in the vicinity of epithelial and EC layers within infected lungs, contrasting with its dispersed distribution in uninfected lungs (73). Moreover, data from *in vitro* experiments demonstrated that Gal-3 acts as an adhesion molecule facilitating direct cross-linking of neutrophils to ECs, a process dependent on Gal-3 oligomerization (73).

Considering data from intravital microscopy and flow chamber shear-based adhesion assays, Gittens et al. propose a dual role for Gal-3, both endogenous and exogenous, in promoting leukocyte migration to inflamed tissues (84). Their report shows that endogenous Gal-3 deficiency impairs leukocyte slow rolling and migration in response to IL-1β, likely due to reduced expression of cell adhesion molecules and changes in cell glycosylation profile (84). Additionally, exogenous administration of Gal-3 triggers the upregulated expression of proinflammatory cytokines and chemokines in the local microenvironment, thereby enhancing the recruitment of neutrophils and monocytes to inflamed sites (84).

Gal-3 also plays a key role in mediating eosinophil trafficking under flow conditions to sites of allergic inflammation (85). Data from studies conducted by Rao et al. show increased expression of Gal-3 in eosinophils obtained from allergic donors (85). They propose that eosinophil adhesion is initially enabled by the binding of eosinophil-expressed Gal-3 and α4β1 integrins to VCAM-1 expressed on ECs. EC-expressed Gal-3 further strengthens this interaction by relocating to the contact points and interacting with eosinophil-expressed Gal-3 and α4β1 integrins (85).

#### 3.1.2 Gal-3-mediated cancer cell adhesion to vascular endothelium

Elevated serum Gal-3 levels in cancer patients have been reported in numerous studies across different types of cancer, including breast cancer (87, 88), colorectal cancer (87), prostate cancer (89), melanoma (90), bladder cancer (91), and lung cancer (92). In some studies, serum Gal-3 levels have been positively correlated with tumor progression, metastasis, and poor prognosis (69, 92, 93). These observations provide a foundation for theorizing that serum Gal-3 is involved in metastasis promotion, likely through supporting adhesion of CTCs to ECs and subsequent extravasation to metastatic sites (94).

Gal-3 is proposed to promote CTC adhesion to EC by binding to Thomsen-Friedenreich (TF) antigen carried by mucin protein 1 (MUC1) expressed on cancer cells, causing patchy redistribution of MUC1 on the cell surface (17, 95). This allows for the exposure of CTC surface adhesion molecules, such as CD44, as well as ligands to endothelial E-selectin, facilitating the interaction with their respective molecular counterparts on ECs (17, 95). The interaction between Gal-3 and cancer-associated MUC4 is also suggested to enhance the binding of tumor cells to ECs in a similar fashion (96). Nevertheless, the adherence of low MUC1-expressing cancer cells to ECs appears to be facilitated by both exogenous and cell-intrinsic Gal-3 through a β-catenin-dependent upregulation of CD44 and N-cadherin expression (97, 98).

Data from investigations led by Chen et al. demonstrate that high levels of Gal-3 enhance cancer cell adhesion to vascular endothelium by stimulating the secretion of various metastatic-promoting cytokines from ECs, such as IL-6 and granulocyte colony-stimulating factor (G-CSF) (99). These cytokines interact with the vascular ECs and cause increased expression of surface adhesion molecules, including αvβ1integrin, E-selectin, ICAM-1, and VCAM-1 (99). Further investigations carried out by the same research team reveal that Gal-3 binds to N-linked glycans on CD146/MCAM expressed on EC, inducing CD146 dimerization and subsequent activation of AKT signaling (100). Additionally, silencing of CD146 expression eliminates the Gal-3-induced secretion of metastatic-promoting cytokines from ECs, namely IL-6 and G-CSF (100).

Wang et al. have recently found that human EC-derived extracellular vesicles (H-EVs) mediate the adhesion of breast cancer cells to human umbilical vein endothelial cells (HUVECs), an effect that is enhanced by circulating Gal-3 (101). Mechanistically, their data show that Gal-3 induces ICAM-1 upregulation in HUVECs, which is transferred to cancer cells via H-Evs, promoting CTC-EC adhesion (101). Interestingly, Zhang et al. have identified Gal-3 as a potential mediator of integrin αvβ1 export into EVs derived from breast cancer cells, promoting their communication with other cell types, including ECs (102). The authors report that knockdown of Gal-3 results in reduced integrin levels on the EV surface and diminished metastatic potential of cancer cells *in vivo* (102).

Noteworthy, Krishnan et al. present evidence of significant constitutive expression of Gal-3 within the vascular ECs of the lungs, indicating that Gal-3 serves as a readily accessible anchoring point for CTCs bearing highly dense Gal-3-binding glycans to promote metastatic colonization of the lungs (86). Their results indicate that highly metastatic melanoma B16F10 cells exhibit upregulated expression of heavily glycosylated Gal-3 receptor ligand, lysosome-associated membrane protein-1 (LAMP-1), compared to the low metastatic melanoma B16F1 cells (86).

#### 3.1.3 Gal-3-mediated stem cell adhesion to vascular endothelium

Mesenchymal stem cells (MSCs) have garnered significant attention over the past two decades for their clinical promise in regenerating damaged tissues and treating inflammatory diseases (103). Their versatility also spans the development of treatments for a wide range of conditions, including degenerative diseases and cancer (104, 105). In-depth comprehension of the dynamics between transplanted stem cells and the host vasculature is crucial for ensuring reliable delivery of stem cells to the targeted locations and maximizing their therapeutic effectiveness (106).

Data on the role of Gal-3 in facilitating stem cell adhesion to ECs is still emerging (107). Results from recent studies conducted by Sedlar et al. show that human adipose-derived stem cells (ADSCs) express an abundance of Gal-3, both inside the cells and on their surfaces, compared to HUVECs (107). Their data indicate that Gal-3-preadsorbed cultivation surfaces enhance the adhesion of both HUVECs and ADSCs, mediated, at least partially, by α5β1, αVβ3, and αVβ1 integrins (107). Interestingly, this adhesion is resistant to blocking with Gal-3 CRD ligand, LacdiNAc (GalNAcβ1,4GlcNAc), suggesting that this interaction is mediated by a mechanism independent of Gal-3 CRD canonical binding with glycoconjugates (107). These findings present a promising avenue for future research and are likely to gain significant attention in the field of vascular tissue engineering and regenerative medicine.

### 3.2 Gal-8

Gal-8 was first detected in rat livers as a 35kDa β-galactoside-binding protein (108). It was also called prostate carcinoma tumor antigen-1 (PCTA-1) due to its high expression on human prostate cancer cells, while absent on normal prostatic tissues and benign prostatic hyperplasia tissues (109). Later, it was revealed that Gal-8 is expressed by various normal cells throughout the body, with vascular and lymphatic ECs considered significant contributors to its production (110).

In humans, the gene encoding Gal-8, *LGALS8*, is located on chromosome 1 (1q42.11), spans 33kbp of genomic DNA, and contains 11 exons (111, 112). Human Gal-8 has at least six isoforms: three isoforms belong to the tandem repeat galectins, while the other three isoforms belong to the proto-type galectins (113). It has been suggested that galectins containing double CRD serve as hetero-bifunctional crosslinking molecules that can recognize distinct glycoconjugates, thus expanding their functional repertoire (98). In this regard, Gal-8 is conceptualized as comprising two distinct terminal domains with different affinities for oligosaccharides: a C-terminal domain (Gal-8C) that preferentially binds non-sialylated glycans such as blood group A and B antigens and poly-N-acetyllactosaminyl glycans, and an N-terminal domain (Gal-8N) which favors sulfated and sialylated glycans (114).

#### 3.2.1 Gal-8-mediated immune cell adhesion to vascular endothelium

Gal-8 orchestrates the interaction between leukocytes, platelets, and vascular ECs, impacting many physiological and pathological processes such as hemostasis, infection, inflammation, and atherosclerosis (115). Data obtained from investigations led by Yamamoto et al. show that Gal-8 triggers marked increases in the adhesion of human peripheral blood leucocytes (T cells, B cells, neutrophils, eosinophils, and monocytes) to HUVECs (116).

Mechanistically, several studies propose that Gal-8 mediates leukocyte adhesion, either to ECs or ECM proteins, through direct interaction with certain members of the integrin family (116). For instance, integrin αM is implicated in modulating Gal-8-induced adhesion of neutrophils to plastic culture plates (117). For studies on human Jurkat T cell line, results obtained by Cárcamo et al. demonstrate that integrin α1β1, α3β1 and α5β1 participate in mediating Gal-8-induced adhesion to immobilized fibronectin (118), whereas investigations carried out by Yamamoto et al. present α4, α5, αL and β1 as the major Gal-8-binding proteins on Jurkat T cells (116).

Moreover, Gal-8 triggers EC activation and induces adhesion of normal platelets, likely through increased von Willebrand factor (vWF) exposure on ECs, which binds to platelet GPIb receptors (115). Gal-8 also stimulates the production of pro-inflammatory cytokines and chemokines within ECs, namely CCL2, CXCL3, IL-8, CXCL1, GM-CSF, IL-6, and CCL5, further exacerbating the inflammatory response (115).

#### 3.2.2 Gal-8-mediated cancer cell adhesion to vascular endothelium

Various immunohistochemical studies have documented a wide variability of Gal-8 expression levels in malignant tissues compared to their normal counterparts, influenced by several factors including organ type, histologic type, and tumor stage (111, 113). However, ELISA-based studies have revealed markedly elevated levels of serum Gal-8 in cancer patients, including colorectal cancer (87), breast cancer (87), multiple myeloma (119), and melanoma (120). Some reports suggest a more pronounced elevation of Gal-8 in serum samples from patients who have developed metastases (87).

Experimental research indicates that elevated levels of circulating Gal-8 potentially facilitate the dissemination of tumor cells by promoting their adherence to vascular ECs (87, 119). Preincubation of colon cancer cells with exogenous Gal-8, at concentrations comparable to those found in sera of cancer patients, results in enhanced cancer cell adhesion to HUVECs monolayer, in a dose-dependent manner (87). This pro-adhesive effect is eliminated in the presence of lactose (87).

Interestingly, Friedel et al. have investigated the impact of two Gal-8 isoforms, Gal-8S and Gal-8L, on the adhesion of myeloma cells to vascular ECs (119). Results derived from this study indicate that both isoforms significantly increase myeloma cell adhesion to ECs under static and dynamic shear stress conditions, with Gal-8L showing a more pronounced effect compared to Gal-8S (119). Moreover, pre-stimulation of ECs with tumor necrosis factor (TNF) and subsequent treatment with Gal-8 leads to a 1.5–1.7-fold enhancement in myeloma cell adhesion compared to TNF-primed cells that are not treated (121). Unexpectedly, data from studies conducted by Gentilini et al. reveal that silencing of Gal-8 in prostate cancer cells impairs homotypic aggregation of cells, with no effect on their adherence to bovine aortic ECs (BAECs) (122). Taken together, these diverse findings suggest a complex role of Gal-8 in cancer cell adhesion to ECs, while also highlighting isoform-specific effects, which remain to be further investigated.

### 3.3 Gal-9

Gal-9, a 36kDa β-galactoside-binding protein belonging to the tandem-repeat type, was first identified in 1997 by three separate research groups (121, 123–125). In humans, the gene encoding Gal-9, *LGALS9*, is located on the long arm of chromosome 17 at locus 11.2 (17q11.2), consisting of 11 exons, in addition to two *LGALS9*-like genes (*LGALS9B* and *LGALS9C*), located on the short arm of the same chromosome at locus 11.2 (17p11.2) (126). Based on the length of the linker peptide, three main isoforms of Gal-9 are described: Gal-9L (large), Gal-9M (medium), and Gal-9S (small), each consisting of 355, 323, and 311 amino acids, respectively, giving Gal-9 further multivalent properties (127). Gal-9 prefers binding to internal LacNAc residues within poly-LacNAc chains and displays a specific affinity for i-linear N-glycans (128). The reactivity of the full-length Gal-9 is independent of sialylation status. However, desialylation results in an increased affinity of Gal-9 N-terminal domain to N-glycans (129, 130).

While Gal-9 is widely distributed in various tissues and variably expressed in the spleen, stomach, colon, lymph nodes, and appendix, studies elucidating the specific cell types within these organs that express Gal-9 remain lacking (131). Gal-9 is known for its immunomodulatory functions and has been studied in various pathological conditions associated with cancer, autoimmunity, graft rejection, inflammatory disease, and microbial infections (132). Importantly, Gal-9 is synthesized and secreted by activated ECs (133), with substantial evidence underscores its functions as an evolving player in EC biology (134). However, the exact role of Gal-9 in regulating circulating cell adhesion to vascular ECs remains to be elucidated (135, 136).

#### 3.3.1 Gal-9-mediated immune cell adhesion to vascular endothelium

Gal-9 was initially described as a potent eosinophil chemoattractant agent in inflammatory processes (137). Although recent publications supporting this notion are deficient, emerging experimental studies have established the pro-adhesive function of Gal-9 on circulating leukocytes and further delved into investigating the underlying mechanisms (135). In line with observations regarding Gal-8, research conducted by Yamamoto et al. indicates that Gal-9 substantially boosts the adhesion of peripheral blood T-cells, B-cells, neutrophils, eosinophils, and monocytes to cultured HUVECs (116). Furthermore, data from studies done by Chakraborty et al. reveal that Gal-9, incidentally expressed at conspicuously high levels on human post-capillary venules, HEVs, and human B cells, facilitates the bridging of human circulating and naïve B cells to human vascular ECs, with concurrent deceleration of transendothelial migration (138). In neutrophils, both soluble and immobilized Gal-9 serve as an adhesion molecule enabling the interaction between neutrophils and ECs through CD44 and β2 integrin-dependent mechanisms (133). Results of a recent study by Mansour et al. point out that, under flow conditions, immobilized Gal-9 promotes the adhesion of CD4+ and CD8+ T cells through glycan and integrin-dependent mechanisms (135). Moreover, data from *in vivo* studies using Gal-9-deficient mice demonstrate impaired leukocyte trafficking and reduced local production of pro-inflammatory chemokines/cytokines (135).

More recently, Gal-9 has been shown to enhance the adhesion of leukocytes to ECs through interaction with blood group H antigen glycans present on EC surfaces (136). This investigation involved a model system utilizing galectin-positive Jurkat cells and EA.hy926 cells (immortalized human umbilical vein ECs expressing blood group H antigen) (136). Results indicate that Gal-9 binding to blood group H antigen facilitates adhesion, with loss of Gal-9 activity in defucosylated EA.hy926 cells, suggesting a potential mechanism for Gal-9-mediated leukocyte-EC interactions (136).

#### 3.3.2 Gal-9-mediated cancer cell adhesion to vascular endothelium

Variation in tumor-intrinsic Gal-9 expression levels has been observed across studies. However, the prevailing trend suggests a low or almost absent Gal-9 in tumor cells compared to their normal counterparts (126). Moreover, Gal-9 expression levels in tumor tissues and cancer cell lines can inversely correlate with tumor progression in various types of cancer, such as breast cancer (139), cervical cancer (140), hepatocellular carcinoma (141), and melanoma (142), yet, high Gal-9 expression can associate with poor clinical outcomes in some other types of cancer (143–145). Conversely, elevated levels of Gal-9 have been reported in sera samples from patients with cancer, including chronic lymphocytic leukemia (146), pancreatic cancer (147), and ovarian cancer (148), with variable correlation with tumor stage and patient survival (146, 148). These observations have prompted speculation regarding a dual role for Gal-9 in mediating tumor metastasis (126). It has been suggested that decreased levels of Gal-9 in tumor tissues compromise cell-cell adhesion and confer anti-apoptotic features to cancer cells, leading to their detachment from the primary site (126). On the other hand, the observed heightened levels of circulating and EC-bound Gal-9 promote CTC-EC interaction, ultimately facilitating tumor cell extravasation and metastasis (126).

Using murine melanoma and colon cancer cell lines, Nobumoto et al. explore the role of Gal-9 in regulating cancer cell adhesion and metastasis (149). Intriguingly, daily i.v. administration of Gal-9 following i.v. inoculation of highly metastatic B16F10 melanoma cells into C57BL/6 mice results in a marked reduction of the number of metastatic nodules in the lung (149). The authors propose that this effect is due to the ability of Gal-9 to block the interaction between adhesion molecules on tumor cells, specifically CD44 and α4β1 integrin, with their respective ligands (hyaluronic acid and VCAM-1) on vascular ECs and ECM, thereby suppressing tumor cell adhesion and metastasis (149).

It is noteworthy that the three variants of Gal-9 modulate the expression levels of E-selectin on colon cancer cells differently, with Gal-9L downregulating E-selectin expression, whereas Gal-9S and Gal-9M upregulate its expression (150). Consequently, they exert varying effects on the adhesion of cancer cells to HUVECs, as evidenced by *in vitro* experiments conducted by Zhang et al. (150). However, future studies are needed to validate these findings in *in vivo* models.

## 4 Therapeutic potential of targeting circulating and EC-bound Gal-3, -8, and -9

There is a rise in research focusing on the development of therapeutic approaches that target galectin-glycan interactions aiming to treat various health conditions (2, 151–155), including inflammatory disorders (156), autoimmune diseases (157), and cancer (158–160). These experimental therapeutics encompass small molecule chemical inhibitors, natural polysaccharides, neutralizing antibodies, and synthetic peptides.

### 4.1 Small molecule inhibitors

The development of small molecule inhibitors against galectin-ligand interactions has concentrated on small β-galactoside derivatives (158). As carbohydrate molecules are rapidly cleared, degraded, and/or metabolized from circulation, identifying an efficacious, long-lasting inhibitor has been challenging (158). Attempts have been made to modify the C1 and C3 positions of β-galactose to improve the interactive profile within the CRD ligand-binding groove (161, 162). Efforts led by Zetterberg et al. to substitute C1 and C3 with chlorophenylthiols and fluoroaryl triazoles have resulted in the development of an α-thiogalactoside derivative for Gal-3, known as GB1107 (163). The orally active Gal-3 antagonist, GB1107, has demonstrated efficacy in attenuating lung cancer growth and metastasis in both human (A549) and mouse (LLC1) cell lines (22). Similarly, studies by Kim et al. have shown that GB1107 inhibits tumor growth in orthotopic mouse models of gastric cancer (164). Moreover, the next-generation orally active analog of GB1107, GB1211 (165), has shown promising results in combination with an anti-PD-L1 blocking antibody, leading to a significant reduction in the growth of LLC1 syngeneic lung cancer models (21). Currently, GB1211 is undergoing assessment in a phase I/II clinical trial (NCT05240131) in combination with the anti-PDL1 monoclonal antibody (mAb) atezolizumab in patients with non-small cell lung cancer (NSCLC) (22).

### 4.2 Natural polysaccharides

Studies conducted by Nangia-Makker et al. employ modified citrus pectin (MCP), a nondigestible, water-soluble polysaccharide fiber derived from citrus fruits, and demonstrate inhibitory effects on the adhesion of breast tumor cells to ECs (166). In other studies, MCP has been shown to blunt melanoma (167), thyroid (168), breast (169), and colon (166) tumor growth, and inhibit spontaneous metastasis of a rat prostate cancer model (170). Likewise, MCP variants, such as PectaSol MCP, PectaSol-C MCP, and GCS-100, have also shown the capacity to impede cancer cell proliferation in *in vitro* experiments (171–173). Another variant, belapectin has shown promise in clinical trials when administered in combination with pembrolizumab for patients with advanced melanoma or head and neck squamous cell carcinoma (NCT02117362 and NCT02575404) (174). However, the use of belapectin as an authentic Gal-3 inhibitor that can directly interact with and inhibit the CRD remains unclear (175).

### 4.3 Neutralizing antibodies

Neutralizing antibodies are emerging as important therapeutic tools for evaluating their inhibitory effect on galectins in disease (176). Neutralizing antibodies offer several advantages over small molecules, such as greater specificity for each galectin species, established pharmacokinetics/pharmacodynamics and bio-distribution knowledge, and exclusive targeting of extracellular galectins (176). Ortega-Ferreira et al. have developed a novel mAb targeting Gal-3, known as E07, for evaluation in a mouse model of systemic sclerosis (177). Data obtained from this study show that E07 significantly reduces the levels of Natural Killer and CD8+ T cells in the bronchoalveolar fluid, indicative of a protective function in damaged lungs (177). Two Gal-9 antibodies, Gal-Nab1 and Gal-Nab2, have been shown to limit the immunosuppressive activity of regulatory T cells (Tregs) and improve anti-tumor immunity (176). Moreover, Gal-Nab1 and Gal-Nab2 partially cross-react with murine Gal-9 and can be used in syngeneic murine tumor model experiments to assess their overall impact on host–tumor immunology (176). Further encouraging data shows that *in vivo* as well as *in vitro* treatment with LYT-200, a humanized anti-Gal-9 mAb developed by PureTech Health, are more effective in killing blood cancer cells compared to conventional treatments (178). The effect of Gal-8 antibodies has also been evaluated by Vicuna et al., revealing that anti-Gal-8 autoantibodies isolated from systemic lupus erythematosus patients can block the interaction of Gal-8 with lymphocyte function-associated antigen-1 (LFA-1), thereby negatively modulating LFA-1 function in the immune system (179).

### 4.4 Peptides

Several peptides targeting galectins have been reported. In studies using MDA-MB-435 breast cancer cells, the use of a T antigen peptide, specifically the Thomsen-Friedenreich antigen-specific peptide (P-30), significantly inhibits cancer cell adhesion to the vascular endothelium (50–74% reduction in a dose-dependent manner) (180). This inhibitory effect is crucial in impeding the early stages of cancer metastasis (180). Similarly, P-30 interferes with the homotypic aggregation of prostate cancer cells (180). Other Gal-3 peptides, G3–C12, have been shown to decrease metastasis formation in breast cancer *in vivo* (181). However, there is a need to explore the selectivity of peptide-based inhibitors towards different galectins and potential off-target effects.

Although galectin inhibitors show great promise as therapeutics, their specificity and selectivity within a particular pathological condition require further exploration. Systemic treatment may result in nonspecific effects on uninvolved organs or physiologies, potentially diminishing treatment efficacy (157). Therefore, employing a more targeted delivery approach to affected tissues and cells could enhance the potential for modulating the effects of galectins *in vivo* without impacting other tissues (157). A deeper understanding of galectin interactions with circulating cells and ECs, coupled with the ability to manipulate these interactions, could greatly enhance therapeutic strategies and improve patient treatment outcomes. Additionally, with their growing role in immunopathologies and cancer, there is a pressing need to develop more potent and selective inhibitors targeting Gal-8 and Gal-9. Nevertheless, targeted galectin therapy holds great promise for enhancing treatment efficacy in patients with inflammatory and autoimmune diseases, as well as cancer.

## 5 Conclusion

**Figure 2:**
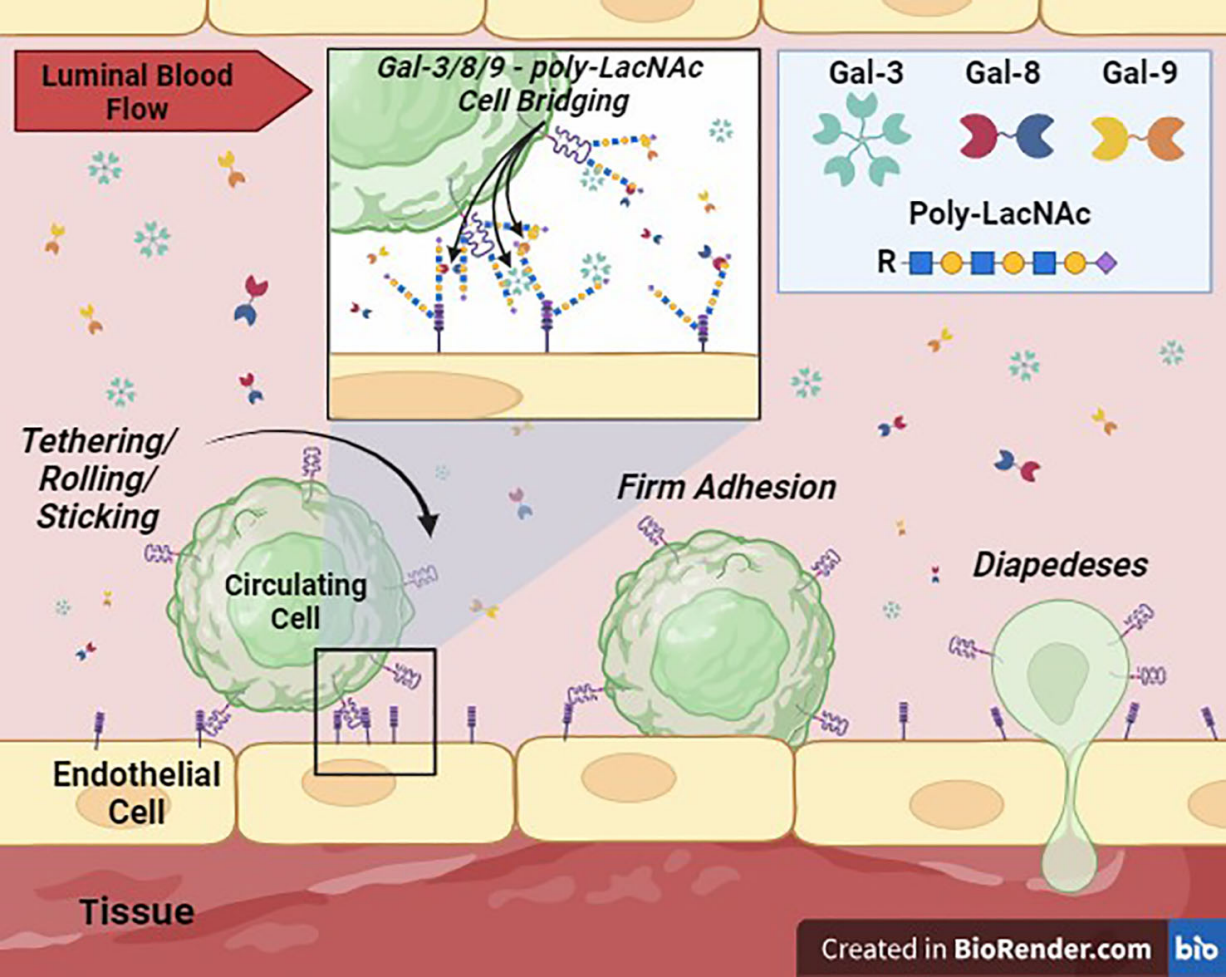
Galectin-Mediated Cell Adhesion to the Vascular Endothelium. This cartoon depicts the proposed critical role of Gal-3, -8, and -9, secreted into the extracellular space (lumen of a blood vessel) potentially by immune, stem, stromal, parenchymal and/or tumor cell sources, in supporting or ‘bridging’ circulating cell adhesion to vascular ECs. Membrane-bound galectins or galectins in solution can theoretically crosslink glycans, such poly-LacNAcs, displayed by surface glycoprotein ligands on cells to mediate intercellular adhesion. These interactions can then facilitate the transition to firm adhesion and diapedesis of cells into tissues.

Extensive research on galectins has unfolded over the past few decades, with emphasis on their pivotal roles in diverse biological/pathobiological processes, including inflammation, immune response modulation, cancer progression, and tissue remodeling. Gal-3, -8, and -9 are emerging as key players in EC biology, contributing significantly to circulating cell entry into tissues via modulation of EC adhesion, vascular permeability, cytokine secretion/regulation, immune cell trafficking, neo/tumor angiogenesis, and tumor metastasis (**Figure 2**). Understanding the multifaceted functions of these galectins in regulating circulating cell adhesion to the vascular endothelium and identifying the underlying mechanisms holds promise for the development of targeted therapeutic interventions for inflammatory and autoimmune diseases and cancer.

## Author contributions

JS: Writing – original draft, Writing – review & editing. NM: Writing – original draft, Writing – review & editing. LL: Writing – original draft, Writing – review & editing. CD: Writing – original draft, Writing – review & editing, Conceptualization, Data curation, Formal analysis, Funding acquisition, Investigation, Methodology, Project administration, Resources, Software, Supervision, Validation, Visualization.
